# Strategies for Identifying and Recruiting Women at High Risk for Breast Cancer for Research Outside of Clinical Settings: Observational Study

**DOI:** 10.2196/54450

**Published:** 2024-09-02

**Authors:** Claire C Conley, Jennifer D Rodriguez, McKenzie McIntyre, Bethany L Niell, Suzanne C O'Neill, Susan T Vadaparampil

**Affiliations:** 1 Department of Oncology Georgetown University Washington, DC United States; 2 Health Outcomes and Behavior Program Moffitt Cancer Center Tampa, FL United States; 3 Department of Diagnostic Imaging and Interventional Radiology Moffitt Cancer Center Tampa, FL United States; 4 Department of Oncologic Sciences University of South Florida Tampa, FL United States

**Keywords:** breast cancer, high-risk populations, risk management, recruitment, woman, women, high risk, observational study, cross-sectional, Facebook, Twitter, flyer, flyers, community events, community event, genetic mutation

## Abstract

**Background:**

Research is needed to understand and address barriers to risk management for women at high (≥20% lifetime) risk for breast cancer, but recruiting this population for research studies is challenging.

**Objective:**

This paper compares a variety of recruitment strategies used for a cross-sectional, observational study of high-risk women.

**Methods:**

Eligible participants were assigned female at birth, aged 25-85 years, English-speaking, living in the United States, and at high risk for breast cancer as defined by the American College of Radiology. Individuals were excluded if they had a personal history of breast cancer, prior bilateral mastectomy, medical contraindications for magnetic resonance imaging, or were not up-to-date on screening mammography per American College of Radiology guidelines. Participants were recruited from August 2020 to January 2021 using the following mechanisms: targeted Facebook advertisements, Twitter posts, ResearchMatch (a web-based research recruitment database), community partner promotions, paper flyers, and community outreach events. Interested individuals were directed to a secure website with eligibility screening questions. Participants self-reported method of recruitment during the eligibility screening. For each recruitment strategy, we calculated the rate of eligible respondents and completed surveys, costs per eligible participant, and participant demographics.

**Results:**

We received 1566 unique responses to the eligibility screener. Participants most often reported recruitment via Facebook advertisements (724/1566, 46%) and ResearchMatch (646/1566, 41%). Community partner promotions resulted in the highest proportion of eligible respondents (24/46, 52%), while ResearchMatch had the lowest proportion of eligible respondents (73/646, 11%). Word of mouth was the most cost-effective recruitment strategy (US $4.66 per completed survey response) and paper flyers were the least cost-effective (US $1448.13 per completed survey response). The demographic characteristics of eligible respondents varied by recruitment strategy: Twitter posts and community outreach events resulted in the highest proportion of Hispanic or Latina women (1/4, 25% and 2/6, 33%, respectively), and community partner promotions resulted in the highest proportion of non-Hispanic Black women (4/24, 17%).

**Conclusions:**

Although recruitment strategies varied in their yield of study participants, results overall support the feasibility of identifying and recruiting women at high risk for breast cancer outside of clinical settings. Researchers must balance the associated costs and participant yield of various recruitment strategies in planning future studies focused on high-risk women.

## Introduction

Breast cancer is the most common non–skin cancer diagnosed among women in the United States [1]. One in 8 US women will develop breast cancer during her lifetime, equivalent to a 13% lifetime risk [1]. However, a subset of women is at high risk for developing breast cancer during their lifetime. Specifically, women who carry a pathogenic variant in genes associated with hereditary breast cancer (ie, *ATM*, *BARD1*, *BRCA1*, *BRCA2*, *CDH1*, *CHEK2*, *NF1*, *PALB2*, *PTEN*, *RAD51C*, and *RAD51D*), who received thoracic radiation between 10 and 30 years of age, or who have an estimated lifetime breast cancer risk of ≥20% are considered to be at high risk for breast cancer [2-4].

Once identified, high-risk women may consider their options for breast cancer risk management, including risk-reducing medication (tamoxifen or raloxifene) and supplemental screening with breast magnetic resonance imaging (MRI) [2-4]. However, uptake of breast cancer risk management options among high-risk women is low. Less than 5% (161/4055, 4.0% for raloxifene; 16/4055, 0.4% for tamoxifen) of eligible women use raloxifene or tamoxifen for breast cancer risk reduction [5], and an estimated 7%-21% (158/2403, 7% observed by Miles et al [6]; 2147/10715, 20% observed by Wernli et al [7]; and 49/228, 21% observed by Ter-Minassian et al [8]) of high-risk women have received a screening breast MRI. Research is urgently needed to understand and address barriers to breast cancer risk management among high-risk women [9].

However, identifying high-risk women for research studies can be challenging. Women at high risk for breast cancer represent a small proportion of the general population; an estimated 1%-15% (5468/42,2406, 1% observed by Miles et al [6]; 25,620/12,30,363, 2% observed by Wernli et al [7]; 183/4266, 4% observed by Weisstock et al [10]; 342/5894, 6% observed by Ozanne et al [11]; 309/2881, 11% observed by Morman et al [12]; and 440/3503, 14% observed by Niell et al [13]) of women presenting for screening mammography are identified as high-risk [6,10-14]. Recruiting high-risk women in clinical settings may be facilitated by routine assessment of breast cancer risk factors [15]. Identifying high-risk women outside of clinical settings presents additional challenges. For example, women may not self-identify as “high-risk” despite the presence of factors that increase their breast cancer risk [16,17] and thus may not respond to study advertisements targeting “high-risk” women. Despite these challenges, there are several significant benefits of recruiting outside of traditional clinical settings. Clinic-based recruitment inherently requires that individuals are connected to the health care system. In contrast, recruiting outside of clinical settings offers the potential for broader reach to groups that have less contact with the health care system [18,19]. Thus, data demonstrating the most effective ways to identify and recruit high-risk women outside of clinical settings would support the recruitment of more diverse, underserved women at high risk of breast cancer in future studies targeting this population.

We successfully recruited women at high risk for breast cancer for an observational study of barriers to screening breast MRI [20,21]. This paper describes recruitment strategies employed for this study and provides metrics (eg, response rate, eligibility rate, cost per eligible participant, and eligible participant demographics) for each strategy.

## Methods

### Procedures and Participants

Eligible participants were assigned female at birth, aged 25-85 years, English speaking, living in the United States, and at high risk for breast cancer. For the purpose of this study, high-risk groups were defined according to criteria from the American College of Radiology (ACR) [4]. Participants were considered at high risk if they had (1) a pathogenic genetic mutation associated with increased risk for breast cancer in self or a first-degree relative; (2) a history of lobular carcinoma in situ (LCIS); (3) received thoracic radiation between 10 and 30 years of age; or (4) an estimated lifetime breast cancer risk of ≥20% per the National Cancer Institute (NCI) Breast Cancer Risk Assessment Tool (BCRAT) [22]. Although other, lengthier risk assessment models (eg, Breast Cancer Surveillance Consortium, Tyrer-Cuzick) have better predictive ability [13,23,24], we chose to use the relatively short BCRAT to estimate lifetime breast cancer risk due to concerns about participant burden.

Exclusion criteria included personal history of breast cancer, prior bilateral mastectomy, medical contraindications for MRI, and not up-to-date on screening mammography per ACR guidelines [25]. Given that our study provided an incentive to participants, we were vulnerable to fraudulent responses by ineligible individuals motivated to harvest the incentive. For this reason, consistent with best practices for detecting fraudulent responses to web-based research studies [26], we excluded individuals who made multiple attempts at completing the web-based eligibility screener. These individuals were identified by IP address, which was captured by our survey platform (Qualtrics) upon submission of the eligibility screener.

**Figure 1:**
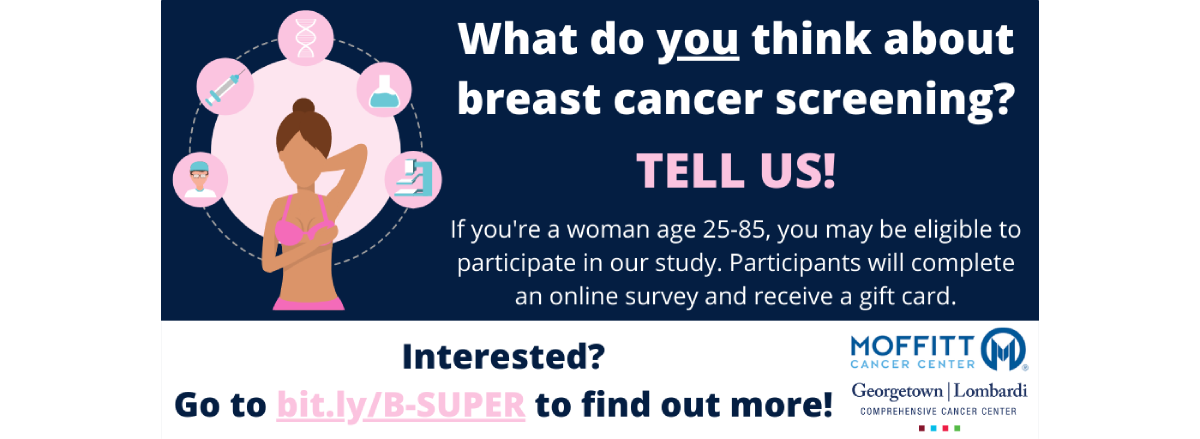
Example study advertisement.

Participant recruitment took place between August 2020 and January 2021. Participants were recruited using a variety of mechanisms including targeted Facebook advertisements, Twitter posts, ResearchMatch, community partners, paper flyers, and community outreach events. Details for each recruitment mechanism are provided in the section titled “Recruitment Strategies.” All advertisements stated that the study was focused on experiences with breast cancer screening, that women aged 25-85 years may be eligible to participate, and that participants would be compensated for survey completion. An example study advertisement is shown in Figure 1. Interested individuals were directed to a secure website (Qualtrics) with eligibility screening questions. Those deemed eligible based on initial screener responses (ie, pathogenic genetic mutation carrier or relative, history of LCIS, or history of thoracic radiation) were able to continue to the web-based survey immediately. For all other respondents, the research team used data elements from the screener to calculate estimated lifetime risk via the NCI BCRAT. Specifically, a member of the research team downloaded Qualtrics data weekly and calculated BCRAT scores for these respondents using the available SAS macro (National Institutes of Health, National Cancer Institute) [27]. Those deemed eligible based on BCRAT scores were emailed a link to the web-based survey.

### Recruitment Strategies

Participants self-reported method of recruitment during the eligibility screener. Response options included targeted Facebook advertisements, Twitter posts, ResearchMatch, community partners, paper flyers, and community outreach events, “word of mouth,” “other,” and “prefer not to answer.”

#### Targeted Facebook Advertisements

Targeted Facebook advertisements were distributed via the Georgetown-Howard Universities Center for Clinical and Translational Science [28]. Facebook allows advertisers to “target” individuals meeting certain demographic characteristics and who express interest in specific topics. For this study, Facebook advertisements were targeted to users: located in the United States, aged 18 years and older, whose language settings were set to English, and whose Facebook “interests” included a breast cancer research or advocacy group (eg, Breast Cancer Research Foundation, Susan G. Komen) or event (eg, National Breast Cancer Awareness Month, Making Strides Against Breast Cancer).

#### Twitter Posts

The study advertisement was distributed via the Georgetown Lombardi Comprehensive Cancer Center Twitter account [29]. Tweets are public by default, meaning that any Twitter user can view and interact with them. Unlike advertisements distributed via Facebook, advertisements posted on Twitter were not targeted to any particular demographic. In addition, these tweets were not “promoted,” meaning that we did not pay for their placement on Twitter. Rather, tweets were displayed to users who “follow” the Georgetown Lombardi Comprehensive Cancer Center Twitter account and other users who the Twitter algorithm deemed may be interested in the topic [30].

#### ResearchMatch

ResearchMatch [31] is a national electronic, web-based research recruitment database funded in part by the National Institutes of Health Clinical and Translational Science Award program and hosted at Vanderbilt University as an institutional review board (IRB)–approved data repository (VUMC IRB #090207). Study staff searched the ResearchMatch database for participants who were female, aged 25-85 years, and who did not report a personal history of breast cancer. Participants meeting these criteria were sent study invitation emails through the ResearchMatch web portal (Multimedia Appendix 1). Interested participants released their contact information to the study team via the ResearchMatch platform. Study staff then followed up via email with a link to the web-based eligibility screener.

#### Community Partners

To further expand our reach, we worked with 2 community organizations serving women at high risk for breast cancer to promote the study. Organization A is a Florida-based nonprofit organization with the goal of supporting people at risk for or living with hereditary cancer in general. Organization B is a nonprofit organization with multiple locations across the United States with the goal of supporting people living with or at increased genetic risk for breast or ovarian cancer specifically. Both organizations distributed information about the study via email and posted about it on social media (ie, Facebook).

#### Paper Flyers

We developed flyers describing the study goals and activities, which included contact information for study staff and a QR code directing interested individuals to the web-based eligibility screener (Multimedia Appendix 2). We mailed copies of the flyer to 279 ACR-accredited mammography clinics within our institutions’ catchment areas. These mailings included a cover letter explaining the purpose of the study and requesting that the flyer be posted in an area where potential participants might view it (ie, waiting rooms).

#### Community Outreach Events

NCI-designated Comprehensive Cancer Centers engage in community outreach and engagement (COE) activities, such as health fairs and other educational events. In the hopes of diversifying our study sample, we worked with COE staff to promote this study at community outreach events, which often target underserved populations (eg, under- or uninsured, racial and ethnic minority groups) within our institutional catchment areas. Specifically, COE staff at both sites brought paper copies of study flyers (described in the section “Paper Flyers”) to distribute at community outreach events.

### Statistical Analysis

All analyses were conducted using SPSS (version 28; IBM Corp). First, descriptive statistics were used to calculate rates of eligible respondents and completed surveys by recruitment strategy. Second, *z* tests were used to compare the proportion of eligible respondents by recruitment strategy. For independent-samples proportions tests, it has been suggested that both events and nonevents should occur at least 10 times in both samples [32]. Thus, we compared only recruitment strategies that identified at least 10 eligible participants and 10 ineligible participants. *z* tests were 2-tailed, and a Bonferroni correction was applied to adjust for multiple comparisons. As 10 *z* tests were conducted, significance was specified as α<.005.

Finally, descriptive statistics were used to characterize the cost per eligible participant (ie, the amount spent on a recruitment strategy divided by the number of eligible participants resulting from that recruitment strategy) and participant demographic information. Cost was defined as direct costs (ie, the amount paid for access to a given recruitment mechanism), personnel costs (ie, the person-hours spent on a given recruitment mechanism multiplied by the hourly staff wage), and overhead costs (ie, fixed costs such as rent, utilities, and phone). Person-hours included time spent on setup (eg, developing flyers and advertisements, meeting with community partners, and posting advertisements on social media), contacting potential participants, screening for eligibility, and following up with eligible participants. Person-hours were estimated retrospectively at the conclusion of the study based on study records of contact with potential participants, conservatively assuming an average of 5 minutes per email contact. The hourly staff wage used to calculate personnel costs was US $23.90 per hour, based on the median wage for social science research assistants in 2021 [33]. Overhead costs were estimated based on our institutional indirect rate in 2021 (56%).

### Ethical Considerations

Study procedures were reviewed by the Advarra IRB (protocol #00000971) and determined exempt from IRB oversight due to minimal risk. The Advarra IRB also provided a waiver of informed consent for this study (protocol #00000971) due to minimal risk. All data were deidentified and participants received a US $10 gift card upon survey completion.

## Results

Between August 2020 and January 2021, we received 1566 unique responses to the eligibility screener (Table 1). The most commonly reported recruitment methods were Facebook advertisements (724/1566, 46%) and emails from ResearchMatch (646/1566, 41%). Recruitment via COE events (17/1566, 1%) and paper flyers (14/1566, 1%) was least commonly reported. Notably, 36 participants reported hearing about the study via “other” methods. “Other” recruitment methods reported included “email” (without any indication of the email’s source), “social media” (without any indication of the specific social media platform), and “private Facebook group.” Interestingly, the “private Facebook group” referred to in open-ended responses was a group for parents of children with ataxia telangiectasia (AT), a rare genetic condition. Parents of children with AT are carriers of a mutation in the *ATM* gene, which is associated with increased risk for breast cancer [34,35]. A member of this group saw our targeted Facebook advertisement and shared it with other group members. This “snowball” recruitment [36] via social media was unplanned and unexpected. In interpreting the results, it is important to note that the “other” category includes this unique source of participants.

While Facebook advertisements yielded the greatest number of eligible respondents (n=258), this represented only 36% (258/724) of respondents recruited via Facebook (Table 1). Although they were associated with fewer eligibility screener responses, the recruitment methods with the greatest proportion of eligible responses were “other” (19/36, 53% eligible), community partners (24/46, 52% eligible), and “prefer not to answer” (7/18, 39% eligible). ResearchMatch had the lowest proportion of eligible respondents, with only 11% (73/646) of respondents being eligible for the study.

Overall, participants were most commonly ineligible because they were not at high risk for breast cancer (702/1162, 60% of ineligible respondents). However, this did vary by recruitment strategy (Table 2). For example, individuals who were recruited via Facebook were most frequently ineligible due to a personal history of breast cancer. In addition, 23% (5/22) of ineligible respondents recruited via community partners and 18% (3/17) of ineligible respondents recruited via “other” methods were ineligible due to a prior bilateral mastectomy. This was much higher than respondents recruited via other methods (0%-5% of ineligible respondents; 0/30 for Twitter, 0/11 for “prefer not to answer,” 0/11 for COE events, 0/11 for paper flyers, 2/573 for ResearchMatch, 7/466 for Facebook, and 1/21 for “word of mouth”). As noted in the section titled “Procedures and Participants,” we excluded individuals who made multiple attempts at completing the web-based eligibility screener, as this is an indicator of fraudulent survey responses [26]. Participants who were recruited via Facebook and ResearchMatch had the highest rates of ineligibility due to multiple attempts (49/466, 11% and 25/573, 4%, respectively).

The eligibility rate for participants identified via ResearchMatch (73/646, 11%) was significantly lower than for participants identified via Facebook (258/724, 36%; *z*=10.50; *P*<.001), community partners (24/46, 52%; *z*=–7.72; *P*<.001), “other” recruitment strategies (19/36, 53%; *z*=–7.09; *P*<.001), and word of mouth (10/31, 32%; *z*=–3.48; *P*<.001) (Table 3). No other recruitment strategies significantly differed in the proportion of respondents who were eligible for the study.

The average age for eligible respondents was 43 years, and the majority were non-Hispanic White (337/404, 83%). However, the demographic characteristics of eligible respondents varied by recruitment strategy (Table 4). For example, participants recruited via ResearchMatch tended to be older than the sample average (mean 45, SD 12.7), while those recruited via Twitter, COE events, and paper flyers tended to be younger than the sample average (mean 36, SD 7.1; mean 34, SD 10.8; and mean 39, SD 7.6, respectively). Although few eligible respondents were identified via Twitter and COE events, these strategies did identify a higher proportion of Hispanic or Latina women than other recruitment strategies (1/4, 25% and 2/6, 33% of eligible respondents were Hispanic or Latina, respectively). The strategy that identified the greatest proportion of non-Hispanic Black respondents was recruiting via community partners (4/24, 17% of eligible respondents were non-Hispanic Black).

Once identified as eligible, participants had the option of continuing on to complete the study survey. The recruitment strategies with the greatest survey completion rates were ResearchMatch (61/73, 84% of eligible respondents completed the survey), “other” (16/19, 84% of eligible respondents completed the survey), and word of mouth (8/10, 80% of eligible respondents completed the survey; Table 1). In contrast, COE events had the lowest rate of survey completion; none of the 6 eligible participants completed the survey.

Three of the recruitment strategies had direct costs: Facebook advertisements, community partners, and paper flyers (Table 5). The other recruitment strategies (ResearchMatch, Twitter, word of mouth, and COE events) incurred personnel costs only. The cost per eligible participant averaged US $105.80 (range: US $3.73 to US $482.71), and the cost per completed survey response averaged US $302.54 (range: US $4.66 to US $1448.13). Word of mouth was the most cost-effective recruitment strategy and paper flyers were the least cost-effective recruitment strategy.

**Table 1:** Respondent eligibility and survey completion rates by recruitment strategy (N=1566).

Recruitment strategy	All responses	Ineligible, n (% of total)	Eligible, n (% of total)	Completed, n (% of eligible)
Facebook	724	466 (64)	258 (36)	124 (48)
ResearchMatch	646	573 (90)	73 (11)	61 (84)
Community partners	46	22 (48)	24 (52)	15 (63)
Other	36	17 (47)	19 (53)	16 (84)
Twitter	34	30 (88)	4 (12)	2 (50)
Word of mouth	31	21 (68)	10 (32)	8 (80)
Prefer not to answer	18	11 (61)	7 (39)	5 (71)
COE^a^ events	17	11 (65)	6 (35)	0 (0)
Paper flyers	14	11 (79)	3 (21)	1 (33)
Total	1566	1162 (74)	404 (26)	232 (57)

^a^COE: community outreach and engagement.

**Table 2:** Reasons for respondent ineligibility by recruitment strategy (N=1162)^a^.

	Total	Facebook	ResearchMatch	Community partners	Other	Twitter	Word of mouth	Prefer not to answer	COE^b^ events	Paper flyers
Not high risk, n (%)	702 (60)	164 (35)	474 (83)	6 (27)	7 (41)	26 (87)	8 (38)	6 (55)	4 (36)	7 (64)
History of breast cancer, n (%)	212 (18)	195 (42)	2 (<1)	6 (27)	1 (6)	0 (0)	4 (19)	3 (27)	1 (9)	0 (0)
Mammogram out of date, n (%)	90 (8)	15 (3)	65 (11)	1 (5)	2 (12)	2 (7)	2 (10)	2 (18)	1 (9)	0 (0)
Multiple attempts at eligibility screener, n (%)	82 (7)	49 (11)	25 (4)	1 (5)	0 (0)	1 (3)	3 (14)	0 (0)	1 (9)	2 (18)
Outside United States, n (%)	44 (4)	26 (6)	5 (1)	3 (14)	3 (18)	1 (3)	1 (5)	0 (0)	4 (36)	1 (9)
Prior bilateral mastectomy, n (%)	18 (2)	7 (2)	2 (<1)	5 (23)	3 (18)	0 (0)	1 (5)	0 (0)	0 (0)	0 (0)
Age <25 years or >85 years, n (%)	8 (1)	5 (1)	0 (0)	0 (0)	0 (0)	0 (0)	2 (10)	0 (0)	0 (0)	1 (9)
Male, n (%)	4 (<1)	3 (<1)	0 (0)	0 (0)	1 (6)	0 (0)	0 (0)	0 (0)	0 (0)	0 (0)
Medical contraindication for MRI^c^, n (%)	2 (<1)	2 (<1)	0 (0)	0 (0)	0 (0)	0 (0)	0 (0)	0 (0)	0 (0)	0 (0)
Total	1162	466	573	22	17	30	21	11	11	11

^a^Percentages indicate proportion of the column total.

^b^COE: community outreach and engagement.

^c^MRI: magnetic resonance imaging.

**Table 3:** Results of z tests for differences in proportion of eligible respondents by recruitment strategy.

	ResearchMatch	Community partners	Other	Word of mouth
Facebook	10.50 (*P*<.001)	–2.26 (*P*=.024)	–2.09 (*P*=.037)	0.39 (*P*=.700)
ResearchMatch	N/A^a^	–7.72 (*P*<.001)	–7.09 (*P*<.001)	–3.48 (*P*<.001)
Community partners	–7.72 (*P*<.001)	N/A^a^	–0.05 (*P*=.957)	1.73 (*P*=.084)
Other	–7.09 (*P*<.001)	–0.05 (*P*=.957)	N/A^a^	1.69 (*P*=.091)

^a^Not applicable.

**Table 4:** Demographic characteristics for eligible participants by recruitment strategy (N=404).

	Age (years), mean (SD)	Race and ethnicity, n (%)							
		NHW^a^	NHB^b^	Latina	Asian	AI/AN^c^	NH/PI^d^	Multiple	Other
Facebook	43 (13.6)	219 (85)	18 (7)	11 (4)	2 (1)	4 (2)	1 (<1)	2 (1)	1 (<1)
ResearchMatch	45 (12.7)	56 (77)	8 (11)	5 (7)	2 (3)	0 (0)	0 (0)	2 (3)	0 (0)
Community partners	43 (11.4)	18 (75)	4 (17)	2 (8)	0 (0)	0 (0)	0 (0)	0 (0)	0 (0)
Other	43 (12.2)	18 (95)	0 (0)	1 (5)	0 (0)	0 (0)	0 (0)	0 (0)	0 (0)
Twitter	36 (7.1)	3 (75)	0 (0)	1 (25)	0 (0)	0 (0)	0 (0)	0 (0)	0 (0)
Word of mouth	44 (9.8)	10 (100)	0 (0)	0 (0)	0 (0)	0 (0)	0 (0)	0 (0)	0 (0)
Prefer not to answer	40 (13.9)	6 (86)	0 (0)	0 (0)	0 (0)	0 (0)	1 (14)	0 (0)	0 (0)
COE^e^ events	34 (10.8)	4 (67)	0 (0)	2 (33)	0 (0)	0 (0)	0 (0)	0 (0)	0 (0)
Paper flyers	39 (7.6)	3 (100)	0 (0)	0 (0)	0 (0)	0 (0)	0 (0)	0 (0)	0 (0)
Total	43 (13.1)	337 (83)	30 (7)	22 (5)	4 (1)	4 (1)	2 (<1)	4 (1)	1 (<1)

^a^NHW: non-Hispanic White.

^b^NHB: non-Hispanic Black.

^c^AI/AN: American Indian/Alaska Native.

^d^NH/PI: Native Hawaiian/Pacific Islander.

^e^COE: community outreach and engagement.

**Table 5:** Cost (in US $) per eligible participant and completed survey response.

Recruitment Strategy	Cost					Cost per eligible participant		Cost per completed survey
	Direct	Personnel	Overhead	Total				
Facebook	1050.00	577.58	911.45	2539.03	9.84		20.48	
ResearchMatch	0.00	2459.71	1377.44	3837.15	52.56		62.90	
Community partners	2000.00	53.78	1134.50	3160.39	131.68		210.69	
Twitter	0.00	87.63	49.07	136.71	34.18		68.35	
Word of mouth	0.00	23.90	13.38	37.28	3.73		4.66	
COE^a^ events	0.00	99.58	55.77	155.35	25.89		—^b^	
Paper flyers	356.68	571.61	519.84	1448.13	482.71		1448.13	
Total	3406.68	3873.79	4061.45	11,314.04	Mean 105.80		Mean 302.54	

^a^COE: community outreach and engagement.

^b^Unable to be calculated, denominator is 0.

## Discussion

### Principal Findings

This paper outlines steps taken in recruitment for a web-based, survey-based observational study of women at high risk for breast cancer. We present data on a variety of different recruitment strategies, including cost-effectiveness. Although recruitment strategies varied in their yield of study participants, our study results support the feasibility of identifying and recruiting high-risk populations outside of clinical settings.

In this study, the overall rate of survey completion among eligible volunteers was 57% (232/404). Although this is comparable with other observational studies in cancer prevention and early detection that have used broad advertising strategies (eg, 191/282, 68% completion among individuals eligible for lung cancer screening recruited via targeted Facebook advertisements [37]), it is significantly higher than previously reported recruitment rates for women at high risk for breast cancer. Padamsee et al [38] described the creation of a community-based cohort of diverse women at high risk for breast cancer. Several different recruitment strategies (including ResearchMatch and targeted Facebook advertising) yielded 3275 eligibility screener responses; of those, 22% (717/3275) were deemed to be eligible, valid, and at high risk for breast cancer. McGuinness et al [39] described strategies for recruiting women at high risk for breast cancer for a randomized controlled trial of a web-based tool to support informed decision-making about chemoprevention. They used a combination of in-clinic and web-based recruitment and reported that only 9% (300/3459) of contacted individuals consented to study participation. The participant yield was much higher (18/54, 33%) among participants recruited online and via posted study flyers but still lower than the yield in our study. One potential explanation for the higher recruitment rate observed in this study is our differing inclusion criteria. Padamsee et al [38] enrolled women with ≥20% lifetime risk according to 1 of 3 risk prediction models (ie, Gail, Claus, and Tyrer-Cuzick). Given their focus on chemoprevention, McGuinness et al [39] sought to recruit women with a history of LCIS or elevated 5-year breast cancer risk (ie, ≥1.67%). Our inclusion of women with pathogenic genetic mutations associated with increased breast cancer risk, who are often highly motivated to participate in research [40], may have bolstered our recruitment rate.

In our study, targeted Facebook advertisements resulted in the most completed survey responses. However, Facebook advertisements were not the most efficient recruitment strategy. Advertising via community partners and “other” recruitment strategies resulted in the greatest proportions of eligible participants as well as high conversion rates to completed surveys. If we consider that participants reporting “other” recruitment strategies included members of Facebook groups for parents of children with AT, both of these recruitment strategies focused on audiences that were enriched with eligible individuals. Identifying forums where eligible individuals may view study advertisements may be one method that researchers can use to increase the efficiency of recruitment in future studies. However, it must be noted that recruiting via community partners was also relatively expensive per eligible participant and completed survey; Facebook advertisements were a cheaper option. Researchers should consider available resources—including financial resources, personnel resources, and time—when selecting strategies for recruiting high-risk women for future studies.

Furthermore, recruiting exclusively via community partners and dedicated support groups may result in samples that systematically differ from the larger population of interest. In order to view study advertisements posted by our community partners, individuals must subscribe to the organization’s mailing list or “follow” them on social media. It is very likely that these individuals not only identify with the organization’s mission but are also highly engaged and informed. Such individuals may also differ from the larger target population in terms of sociodemographic characteristics. Web-based recruitment for research studies can result in samples that are less racially and ethnically diverse than samples recruited using traditional methods [41]. As has been observed in prior studies, our sample was primarily non-Hispanic White. Yet web-based recruitment does not necessarily mean that resulting samples will be demographically homogenous. The subsample of high-risk women recruited on the web by McGuinness et al [39] was 29% (15/51) non-Hispanic Black and 27% (14/51) Hispanic or Latina. Padamsee et al [38] recruited high-risk women online, 35% (251/717) of whom were Black. Therefore, it is possible to identify and recruit racially and ethnically diverse high-risk women via web-based strategies. However, strategies for advertising must be carefully developed, preferably with input from members of the target group(s), and specific resources must be allocated for advertising and recruitment [42,43].

Unexpectedly, the recruitment strategy with the lowest rate of eligible participants was the National Institutes of Health–sponsored registry ResearchMatch. The proportion of eligible participants identified via ResearchMatch was significantly lower than those identified via Facebook advertisements, community partners, “other” methods, and word of mouth. The primary reason for ineligibility of participants identified via ResearchMatch was that they were not at high risk for breast cancer as defined by this study (474/573, 83% of ineligible respondents). Unlike other recruitment strategies (eg, targeted Facebook advertisements and community partners), ResearchMatch did not allow us to focus on users with specific interests; we were able to specify only the age range and medical conditions of the participants. Therefore, it is likely that we reached a more general audience with ResearchMatch than other recruitment strategies. However, ResearchMatch was associated with one of the highest rates of survey completion among eligible participants, perhaps reflecting the high level of motivation to participate in research among individuals who have chosen to register for a research participant database. In short, recruiting via ResearchMatch required our team to screen nearly 600 ineligible participants, incurring personnel costs of about US $53 per eligible participant and about US $63 per completed survey response. Again, this highlights how researchers must balance cost, effort, and potential yield of various recruitment strategies.

Taken together, these results suggest that respondents identified through different recruitment mechanisms effectively represent different subpopulations. For example, respondents who heard about the study via Facebook were most likely to have a personal history of breast cancer. In contrast, respondents who heard about the study via ResearchMatch and Twitter were most often at average risk for breast cancer. Regarding demographic characteristics, individuals recruited via COE events and community partners were more racially and ethnically diverse than respondents recruited via other methods. These patterns shed light on which recruitment methods would be most effective for reaching which groups, akin to audience segmentation approaches that are ubiquitous in marketing research and have been increasingly applied in health settings [44,45]. By identifying the unique characteristics of population segments, future research can focus recruitment resources on specific subpopulations of interest.

The results presented here must be interpreted cautiously in light of limitations. First, we did not track the number of personnel hours associated with each recruitment strategy during the recruitment period but estimated them retrospectively at the end of the study. Thus, our estimate may over- or underrepresent the number of person-hours associated with using various recruitment platforms. In future studies, personnel time should be tracked during the recruitment period in order to accurately estimate the costs associated with recruitment. Relatedly, we estimated overhead costs based on our institutional indirect rate. This is an approximate cost of conducting this study in our setting, which is fairly generalizable to other academic and nonprofit settings, but may not be generalizable to all business contexts. Second, although the study team reviewed each survey response for validity, it is possible that some of the survey responses were fraudulent [26]. This is particularly common in studies that provide compensation for survey responses, such as this one. Future studies might incorporate additional methods to ensure data integrity, such as attention checks or CAPTCHA algorithms [26,46,47]. Third, given that recruitment occurred outside of clinical settings, breast cancer risk factors were assessed via self-report. Our assessment of eligibility thus may be subject to reporting biases. Relatedly, lifetime risk—a key eligibility criterion—was estimated using the relatively short NCI BCRAT due to concerns about participant burden [22]. Other, more in-depth risk assessment models have better predictive ability [13,23,24] and might be used in future research. Fourth, results cannot be generalized to breast cancer survivors, who may be at high risk for breast cancer, but were excluded from this study. Fifth, data were collected during the COVID-19 pandemic, which significantly affected recruitment of participants for research studies. For example, our community partners adjusted their activities and priorities in response to the emergent pandemic-related needs. Similarly, our institution was restricted in the types and frequency of community outreach events that could be hosted. We also attempted to recruit participants via paper flyers in mammography clinic settings, but receipt of cancer screening significantly declined during the early months of the COVID-19 pandemic [48,49]. While rates of screening mammography rebounded by mid-2021 [50], it is still unclear whether the results presented here would generalize outside of the acute pandemic timeframe, and replication of this study may be needed. Finally, conclusions about the effectiveness of recruitment strategies were drawn based on proportion of eligible respondents and the number of participants who ultimately completed the study survey; we did not capture individuals’ perspectives on advertising materials or strategies, nor the proportion of responders versus nonresponders among individuals who viewed the advertisements. Future studies are needed to more thoroughly evaluate barriers and facilitators to recruitment of high-risk women for research studies.

### Conclusions

Using a variety of web-based and in-person methods, we successfully identified and recruited women at high risk for breast cancer outside of clinical settings, supporting the feasibility of recruiting and retaining this unique population for participation in behavioral research. However, additional research is needed to identify best practices for recruiting a more demographically diverse group of high-risk women. Although our study focused on women at high risk for breast cancer, results may also provide insight into identification and recruitment of other high-risk populations eligible for risk-based cancer screening (eg, lung cancer screening). Researchers seeking to recruit individuals at high risk for cancer may choose from a variety of recruitment strategies but must balance the associated costs and participant yield.

## Appendix

Multimedia Appendix 1 ResearchMatch recruitment email.

Multimedia Appendix 2 Example study recruitment flyer.

## Abbreviations

ACR: American College of Radiology
AT: ataxia telangiectasia
BCRAT: Breast Cancer Risk Assessment Tool
COE: community outreach and engagement
IRB: institutional review board
LCIS: lobular carcinoma in situ
MRI: magnetic resonance imaging
NCI: National Cancer Institute

## Data Availability

Data are publicly available in the Open Science Framework [51].
