# Supplementary material for: Strategies for Identifying and Recruiting Women at High Risk for Breast Cancer for Research Outside of Clinical Settings: Observational Study
Source: J Med Internet Res. 2024 Sep 2;26:e54450. doi: 10.2196/54450 (PMC11406107; doi:10.2196/54450)
Supplement: Multimedia Appendix 1 [file jmir_v26i1e54450_app1.pdf]

From: ResearchMatch <info@researchmatch.org>

researchmatch.org

Difficult diseases have met their match.

A research team with Georgetown University in Washington, DC, believes you might be a good match for the following study:

Would you like to help researchers at Georgetown University understand why women do or do not participate in breast cancer screening?

Breast cancer screening can detect breast cancer in its early stages, when it is most treatable. However, many women do not get recommended breast cancer screening. Researchers at Georgetown University are interested in learning more about what factors are important to women when they are deciding whether or not to get breast cancer screening.

You may be eligible for this study if you:

- Are female
- Are between 25-85 years of age
- Can read and speak English
- Have NOT been diagnosed with breast cancer

Women who have never had breast cancer screening may still participate.

If you join this study, you will complete a survey designed to ask you about your opinions about and experiences with breast cancer screening. The survey will take between 30-45 minutes to complete, and will be completed online. You will receive a $10 Visa gift card in appreciation of your time and effort.

If you are interested in this study and having the research team contact you directly, please select the "Yes, I'm interested" link below. By clicking the "Yes, I'm interested" link, your contact information will be released to the research team. If you select the "No, thanks." link or do not respond to this study message, your contact information will not be released to the research team.

Yes, I'm interested! **No**, thanks.

Thank you for your interest in ResearchMatch.

ResearchMatch Disclaimer
You are receiving this email message since you have registered in the ResearchMatch registry. Should you wish to edit your profile please click here to login and update your profile.

ResearchMatch is a free and secure tool that helps match willing volunteers with eligible researchers and their studies at institutions across the country. ResearchMatch is only providing a tool that allows you to be contacted by researchers about their studies. ResearchMatch therefore does not endorse any research, research institution, or study. Any recruitment message that you may receive about a study does not mean that ResearchMatch has reviewed the study or recommends that you consider participating in this study.

If you no longer wish to be part of ResearchMatch, please remove your account by clicking here.
