# Supplementary material for: Strategies for Identifying and Recruiting Women at High Risk for Breast Cancer for Research Outside of Clinical Settings: Observational Study
Source: J Med Internet Res. 2024 Sep 2;26:e54450. doi: 10.2196/54450 (PMC11406107; doi:10.2196/54450)
Supplement: Multimedia Appendix 2 [file jmir_v26i1e54450_app2.pdf]

# Has your family member had breast cancer?

## You may be eligible to participate in a research study to share your opinions and experiences with breast screening!

People of all genders who need breast cancer screenings are potentially eligible, including transgender men and non-binary people with breast tissue.

You may be eligible if you have NEVER been diagnosed with breast cancer and are able to speak English.

Someone with a family history of breast cancer may be eligible for breast screening with magnetic resonance imaging (MRI). We would like you to share your opinions and experiences of breast screening.

Your participation could lead to the development of programs designed to help those in the community who are living with elevated risk for breast cancer.

# Next Steps

Individuals who have never had a breast MRI may still participate.

You will be asked to complete a brief survey that will take about 30 minutes.

Upon completion, participants will receive compensation with a gift card.

# Interested?

**Contact Us!**

**E-mail:** bsuper@georgetown.edu
**Phone:** (202) 784-2202
**Web:** bit.ly/B-SUPER
**Scan** this QR code!

Georgetown | Lombardi COMPREHENSIVE CANCER CENTER

MOFFITT CANCER CENTER
